# Coexistence of risk factors for cardiovascular diseases among Brazilian adolescents: Individual characteristics and school environment

**DOI:** 10.1371/journal.pone.0254838

**Published:** 2021-07-19

**Authors:** Thales Philipe Rodrigues da Silva, Fernanda Penido Matozinhos, Lucia Helena Almeida Gratão, Luana Lara Rocha, Luisa Arantes Vilela, Tatiana Resende Prado Rangel de Oliveira, Cristiane de Freitas Cunha, Larissa Loures Mendes

**Affiliations:** 1 Pediatrics Department, Universidade Federal de Minas Gerais, Belo Horizonte, Minas Gerais, Brazil; 2 Department of Maternal and Child Nursing and Public Health, Universidade Federal de Minas Gerais, Belo Horizonte, Minas Gerais, Brazil; 3 Department of Preventive and Social Medicine, Universidade Federal de Minas Gerais, Belo Horizonte, Minas Gerais, Brazil; 4 Nutrition Department, Universidade Federal de Minas Gerais, Belo Horizonte, Minas Gerais, Brazil; 5 Nutrition Department, Pontifícia Universidade Católica de Minas Gerais, Belo Horizonte, Minas Gerais, Brazil; University of Southern Queensland, AUSTRALIA

## Abstract

Cardiovascular diseases (CVD) share common and modifiable risk factors; among them, unhealthy eating, physical inactivity, alcohol intake and smoking habit. However, these factors are not observed in separate and, most often, they influence each other. Risk factors established during adolescence are highly likely to remain in adult life. The aims of the current study were to evaluate the prevalence and coexistence of risk factors for CVD, as well as to investigate individual characteristic of the adolescent and environmental factors associated with risk factors’ coexistence profiles. This was a cross-sectional, national, school-based epidemiological study that estimated the prevalence of cardiovascular risk factors and metabolic syndrome in adolescents aged 12 to 17 years who attended public and private schools located in Brazilian counties with a population of more than 100 thousand. For this study, thematic blocks referring to alcohol consumption, eating habits, smoking, and physical activity were used. The grade of membership method was used to identify the coexistence of risk and protective factors for CVD among adolescents. The study analytical sample comprised 71,552 adolescents. Multilevel logistic regression was used to assess the association between factors influencing the coexistence profile of risk factors for CVD. Based on adolescent-level variables, has shown that meeting positive criterion for Common Mental Disorders and not consuming the meals provided by the school have significantly increased the likelihood of belonging to the CVD-risk profile. On the other hand, school-level variables, show that studying in private schools and living in economically favored Brazilian regions have increased adolescents’ likelihood of belonging to the CVD-risk profile. These results can be used to substantiate the inclusion of food environment variables in public policies focused on preventing CVD development among Brazilian adolescents.

## Introduction

According to estimates, 17.8 million people have died due to Cardiovascular diseases (CVDs) in 2017, which is the main cause of death worldwide [1,2]. Mortality rates associated with this condition in low- and middle-income countries remained high from 1990 to 2017 (368.2 and 316.9 per 100,000 deaths, respectively) [1].

CVDs share common and modifiable risk factors [1,3–5]; among them, unhealthy eating, physical inactivity, alcohol intake and smoking habit, all together, account for approximately 70% to 80% of CVD cases worldwide [3]. Although the most severe manifestations, such as acute myocardial infarction and stroke, are more prevalent in adulthood, risk factors for CVD have been often observed in children and adolescents. These factors emerge early and present high prevalence among Brazilian adolescents [6–9] and among adolescents from other countries [10]. Risk factors established during adolescence are highly likely to remain in adult life [11,12].

The individual evaluation of risk factors for CVD among adolescents provides disease prevalence estimates and helps better understanding how they emerge [6–9]. Comumente estes fatores de risco para as DCV são avaliados separadamente, sem considerar a possibilidade de coexistirem e se influenciarem mutuamente na determinação do desfecho nas DCV [13]. However, these factors are not observed in separate [14,15] and, most often, they influence each other [13]. Thus, it is important taking into consideration the likely coexistence of risk factors for CVD among adolescents.

Therefore, adolescence is a critical stage for the development CVD risk factors [16]. Furthermore, the incidence of two, or more, risk factors during adolescence is enough to predict cardiovascular events within the following 10 years. The combination of such factors, which prevail in adulthood, increases the extent and severity of vascular lesions [16].

Thus, it is essential assessing CVD risk factors at this developmental stage, by taking into consideration their coexistence, rather than approaching them in separate. Grouped patterns of health-related risk behaviors often appear in adolescence [17–20] and the coexistence of these risk factors, mainly for CVD, can increase the number of unfavorable outcomes [21]. Thus, it is necessary conducting analyses focusing on the correlation between these factors to enable performing more effective interventions based on multiple components. It must be done in order to reverse and reduce the number of early risk factors in adolescence, since the combination of two, or more, risk factors is often associated with increased risk of developing cardiovascular diseases [22].

Therefore, the aims of the current study were to evaluate the prevalence and coexistence of risk factors for CVD, as well as to investigate individual characteristic of the adolescent and environmental factors associated with risk factors’ coexistence profiles.

## Materials and methods

### Study design, population and data collection

Survey based on data deriving from the “Study about Cardiovascular Risks in Adolescents” (ERICA—Estudo de Riscos Cardiovasculares em Adolescentes), which was conducted from March 2013 to December 2014. ERICA is a national, cross-sectional, school-based epidemiological study focused on estimating the prevalence of cardiovascular risk factors and metabolic syndrome in 12-to-17-year-old adolescents enrolled in public and private schools in Brazilian cities with more than 100 thousand inhabitants. ERICA sample is representative of medium- and large-sized counties (≥ 100 thousand inhabitants) at national and regional level, as well as of Brazilian capitals [23].

The herein investigated population was stratified into 32 strata comprising 27 Brazilian capitals and 5 sets of counties with more than 100 thousand inhabitants in each of the 5 geographic macro-regions in the country. ERICA study’s sample comprised 12-to-17-year-old male and female adolescents, who were enrolled in the last three years of elementary school, as well as in all three years of high school, in the morning and afternoon shifts, in public and private schools [23].

Schools in each geographic stratum were selected based on probability proportional to size and inversely proportional to distance from the capital—it resulted in the total number of 1,251 eligible schools. Schools distributed in 273 Brazilian counties accounting for more than 100 thousand inhabitants on July 1^st^, 2009 (124 counties, in total) were taken into consideration. A survey of classes and students enrolled in the investigated grades was carried out to enable selecting three classes per school, based on different combinations of shifts (morning and afternoon) and grades (seventh, eighth and ninth grade of Elementary School and first, second and third year of high school). The sample was featured as complex; therefore, calibrated sample weights and correction factor [23,24] were calculated. Evening classes were not taken into consideration due to logistical and operational issues. All students in the selected classes were invited to participate in the study [23].

ERICA’s sample was calculated by taking into consideration 4% prevalence of metabolic syndrome in adolescents, with maximum error of 0.9% and at 95% confidence level. There was increase by 15% in *n* initially calculated to compensate for expected losses and non-response. Thus, the total sample was estimated at 74,628 adolescents, and it was rounded down to 75,060 adolescents [23].

Adolescents in age groups other than 12 to 17 years, who presented disability capable of preventing them from undergoing the anthropometric assessment and from filling out the questionnaire, as well as pregnant adolescents, were not eligible to participate in the study. Detailed information about sampling process, research protocol and data collection were described by Bloch et al. [24] and Vasconcellos et al. [23].

ERICA sample comprised 102,327 eligible adolescents: 73,160 of them completed a 24-hour food record and 74,589 completed the self-administered questionnaire by using personal digital assistant, model LG GM750Q (which comprises 100 questions divided into 11 blocks that cover sociodemographic, health and lifestyle aspects). Therefore, 71,552 adolescents presenting complete data for the adolescent’s questionnaire and 24-hour food record were evaluated. Most adolescents who did not participate in ERICA were male in the age group 15–17 years. In addition, these point estimates assumed that ERICA participants represented individuals who did not participate in the study [23].

The current study has evaluated 71,552 adolescents from 1,247 schools in 124 Brazilian counties.

### Variables’ description

Variables presented in Chart 1 were used to assess the prevalence and coexistence of risk factors for CVD.

**Chart 1:** Indicator of risk factors for cardiovascular diseases.

Risk factor	Assessment in the survey	Adopted definition
*Alcohol intake* [6]	Having consumed at least 1 glass (250 ml) of alcoholic beverage in the last 30 days	0 “never drank alcohol”; 1 = “only once”, denoted “don’t drink”; 2 = "1 or 2 days", 3 = "3 to 5 days", 4 = "6 to 9 days", 5 = "10 to 19 days", 6 = "20 to 29 days", or 7 = “>29 days”, denoted “every day”
*Smoking habit* [7]	Question about the frequency of smoking (number of days) in the previous month	Adolescents who had smoked cigarettes at least 1 day throughout the previous 30 days were considered current cigarette smokers.
Physical activity [8]	Total physical activity time was calculated based on the sum of the time spent in each activity, including the low-intensity ones, such as walking the dog and taking care of children; commuting; and walking to school, home, or work.	Adolescents who did not accumulate at least 300 min/week of physical activity were considered inactive at leisure time [25]
*Ultra-processed food (UPF) intake*	Food intake was assessed based on 24-hour recall (R24h) during face-to-face interview carried out by trained interviewers.	Excessive UPF intake was defined as UPF intake higher than, or equals to, the 80th percentile of the distribution of intake UPF (45.60% of TCV). Large quintile of UPF intake distribution (P80) was associated with poor food intake profile and with high risk of obesity in previous studies [26].

Smoking habit was measured based on definitions adopted by the World Health Organization and by the American Center for Disease Control and Prevention (CDC) in the Global Youth Tobacco Surveillance (GYTS) [18].

The criterion set by WHO was used to assess the leisure-time physical activity level. The product between time and frequency in each activity was calculated, as well as the sum of recorded times [19].

Ultra-processed food (UPF) intake, as well as caloric and food intake, were analyzed and classified according to the NOVA classification system [27], based on the extent and purpose of food processing in fresh or minimally processed, processed and ultra-processed food. Information about the rate of contribution to total daily energy intake (% of total caloric value (TCV)) by the UPF group was taken into consideration in the current study. Ingredients observed in UPF often comprise substances and additives, such as sugar, oil, fat, salt, antioxidants, stabilizers and preservatives [27].

Variables likely capable of influencing the coexistence of risk factors for CVD were divided into two different levels. The first level comprised the following sociodemographic variables: sex (male and female), age in full years (12 to 13 years, 14 to 15 years and 16 to 17 years), self-reported skin color (white, black and Asian-like color), person(s) with whom participants live (both parents, at least one parent or alone), paid work in the last 12 months (no and yes) and maternal education (major degree, complete high school, complete elementary school, incomplete elementary school or illiterate). The habit of eating food provided by schools and the incidence of common mental disorder (CMD) were also taken into consideration at this level. CDM assessment was based on Goldberg’s General Health Questionnaire (GHQ), which was validated for the Brazilian population [22]. Binary system with cut-off point 5 was used in this assessment, i.e., CMD incidence was defined when at least 5 out of 12 items were answered through one of the last two options (“a little more than usual” or “much more than usual”). This criterion presented 73% sensitivity, 90% specificity and 61.2% positive predictive value [23].

School environment variables were the ones evaluated at the second level, namely: school location (capitals or non-capitals) and whether they were located in the most or lesser economically favored regions (most favored regions—South, Southeast and Midwest; lesser favored regions—North and Northeast), school administration type (public or private), whether they sold any food type in their premises (no or yes) and whether the school provided meals to students (no or yes).

### Statistical analysis

#### Grade of membership (GoM)

GoM [28] was used to identify the coexistence of risk and protective factors among adolescents. Diffuse pertinence is allowed in this method, which is used to estimate scores to be attributed to degrees of relevance for each individual in different sets. The model is applied to data set comprising *i* individuals (i = 1, 2,…, I), with *j* categorical variables (j = 1, 2,…, J). There are L_j_ response levels for each *j*-th variable. Discrete response variable ‘X_ijl_’ is predicted by two sets of generated coefficients, namely: λ_kjl_ and g_ik_; wherein λ_kjl_ is the likelihood of attribute incidence between pure profile types to assume any value between 0 and 1. The model estimates the score attributed to the degree of pertinence (g_ik_) for each individual, which represents the degree to which element *i* belongs to the extreme profile, and ranges from 0 to 1–100% degree of pertinence corresponds to extreme profiles (k) [28].

Preponderance criterion, which is the λ_kjl_/marginal frequency ratio (Expected/Observed Ratio), establishes an objective criterion for the profile featuring the generated extremes. Marginal frequency can be understood as the likely incidence of a given feature in the total population. Based on cutoff value of 20%, the likely incidence of *l*-th response to *j*-th variable in *k*-th profile among pure types of that profile must be at least 20% higher than the observed marginal likelihood [28,29]. Risk factors coexisted in the current study when there were at least two risk factors for CVD in the generated profile [20].

The number of extreme profiles k was predetermined for each GoM round. Analysis was performed based on six different models (k = 2, 3, 4, 5, and 6 profiles). Akaike information criterion (AIC) was used to define the most appropriate representation model (tested up to k = 6) [30]. The decision rule corresponds to the model presenting the minimum AIC statistical value. The g_ik_ and λ_kjl_ parameters were estimated in the GoMRcpp.R software for R [31].

After the profiles were created and the GiK of each teenager was found, they were separated based on the highest degree of belonging to the profile. They were categorized as belonging to profile 1 when GiK was ≤ 0.5 and, as belonging to profile 2, when GiK was > 0.5.

The prevalence of extreme profiles in the analyzed population was calculated as follows:

$$P_{k}=\frac{\sum_{i=l}^{l}g_{ik}}{\sum_{i=l}^{l}i}\;with\;k=1,2,\ldots ,K.$$

Such a prevalence can be considered a weighted average because the weight corresponds to the proportion of the population that does not show degree of relevance to the referred profile higher than 0, as well as lower than, or equal to, 1.

#### Multilevel model

Multilevel logistic regression was used to assess the association between factors influencing the coexistence profile of risk factors for CVD. Multilevel analysis has taken into consideration multiple aggregation levels in its estimates—which made Standard Errors (SE), Confidence Interval and hypothesis tests more accurate [28,29].

The modeling process has followed the steps suggested by Laros and Marciano (2008) [28] and it encompassed 3 stages. Stage 1 comprised the Null Model (M0) and estimated the random effect of the model’s intercept. Multilevel logistic regression models were subjected to bivariate analyses to enable selecting individual variables in the null model. P value ≤ 0.20, found in bivariate analysis, was used as variable-inclusion criterion to build the multilevel logistic model with individual variables.

Stage 2, called the Fixed Effects Model, has analyzed the model comprising individual-level variables. Subsequently, stage 3, which was called the Random Effects Model, comprised the inclusion of school-level and individual-level variables in the model. Variance reduction was calculated at the end of the modeling process, based on the introduction of individual-level variables in the models in order to check model’s fit [28]. Variance Partition Coefficient (VPC) was calculated to investigate the proportion of total variance attributed to schools. Akaike information criterion (AIC) was used to calculate model’s fit—the best fit corresponded to the lowest value recorded for this criterion [30].

Gllamm command was used to perform the multilevel aggregated analysis model, which allowed making statistical analysis by taking into consideration the multilevel structure of data, as well as including the weighting necessary to analyze complex samples. Adolescent’s school was used as aggregation unit.

All analyses adopted 5% significance level. OR and 95% CI were used as measures of association. Collected data were analyzed in Stata software, version 14.0.

#### Ethics approval and consent to participate in the study

The study was approved by the Research Ethics Committees of the institution coordinating the study (IESC/UFRJ) and of each Brazilian state. Adolescents who agreed to participate in the study have signed the written informed consents form; parents or legal guardians provided written informed consents form for all participants younger than 18. Participants’ identification remained confidential.

## Results

The present study has evaluated 71,552 adolescents: 55.47% were girls, 63.50% self-declared to be non-white, 37.27% were in the age group 14–15 years, 78.50% were enrolled in public schools and 54.83% lived with both parents (Table 1).

**Table 1:** Features of Brazilian adolescents evaluated through ERICA study. Brazil, 2013–2014. (n = 71,552).

Variable	Absolute Frequency	Relative Frequency (%)
*Gender*		
Female	39,690	55.47
Male	31,862	44.53
*Race/Ethnicity*		
White	25,425	36.50
Non-white	44,225	63.50
*Age (years)*		
12–13	19,832	27.72
14–15	26,670	37.27
16–17	25,050	35.01
*School management type*		
Public	56,168	78.50
Private	15,384	21.50
*Residence*		
Living with both parents	39,231	54.83
Living with at least one parent	27,402	38.30
Living alone	4,919	6.87

Table 2 presents the prevalence and λkjl coefficients of CVD risk factors, which were generated for each pure profile attributed to the evaluated adolescents. Six profiles (k = 6) were generated; the one presenting k = 2 recorded the lowest AIC value in all GoM analyses.

**Table 2:** Distribution of lambda coefficients (λ_kjl_) of internal variables for each extreme profile of Brazilian adolescents’ behavior patterns–ERICA, Brazil.

	n(%)	Profile 1 λ1_jl_(E/O Ratio)	Profile 2 λ2_jl_(E/O Ratio)
**RISK FACTORS FOR CARDIOVASCULAR DISEASES**			
**Smoking habit**			
No	70,064(97.92)	1.0000(1.0212)	0.9274(0.9471)
Yes	1,488(2.08)	0.0000(0.0000)	0.0726(**3.4910**)
**Alcohol intake**			
No	54,131(75.65)	1.0000(**1.3218**)	0.0000(0.0000)
Yes	14,905(20.83)	0.0000(0.0000)	0.8741(**4.1961**)
No information	2,516(3.52)	0.0000(0.0000)	0.1259(**3.5804**)
**UPF intake**			
<80^th^ percentile of the distribution of UPF intake	57,242(80.00)	1.0000(**1.2500**)	0.4489(0.5611)
≥80^th^ percentile of the distribution of UPF intake	14,310(20.00)	0.0000(0.0000)	0.5511 (**2.7556**)
**Practice of physical activity**			
Active (≥300 min/week)	31,770(44.40)	0.41327(0.9317)	0.5112(1.1513)
Inactive (<300 min/week)	39,782(55.60)	0.5863(1.0545)	0.4888(0.8792)

Adolescents presenting total degree of belonging (g_ik_ = 1) to profile 2, i.e., the ones who belonged 100% to the profile, have shown behavioral characteristics such as smoking habit, alcohol intake and UPF intake (≥ 80.00% of TCV). This profile was categorized as risk of CVD development, because it comprised three risk factors. The weighted prevalence of the CVD-risk profile was 29.53%.

The null model is shown in Table 3. The intercept variance (0.41; 95% CI 0.40–0.42) in M0 has shown that the degree of belonging to the CVD-risk profile differed among schools (p<0.001). Variance Partition Coefficient (VPC) reached 0.067, or approximately 6.70% of total variance was attributed to the characteristics of the schools of adolescents.

**Table 3:** Multilevel logistic regression model (OR and p-value) without explanatory variables–Null model.

	Model (M0)–Null Model	
	OR(CI95%)	p-value
**Fixed Effect**		
Intercept	0.41(**0.40–0.42**)	**<0.001**
**Random effect**		
Variance (standard error)	0.23(0.003)	
Variance Partition Coefficient (VPC)	0.067	
AIC	1292760	

“Belonging to the CVD-risk profile” is a factor directly associated with boys in the age groups 14–15 years and 16–17 years, self-referred as black and mixed skin color, who lived with at least one parent or alone, who have had a paid job in the previous 12 months, presented positive CMD criterion, consumed food provided at school, and whose mother had low schooling.

With respect to schooling level, factor “belonging to the CVD-risk profile” was directly associated with private schools located in capital cities, in economically favored Brazilian regions, where food was sold inside or around the school (Table 4).

**Table 4:** Bivariate analysis based on multilevel logistic regression model (OR and p-value) of individual characteristic of the adolescent and school environment according to profiles generated for coexistence of risk factors for cardiovascular diseases among Brazilian adolescents. –ERICA, Brazil, 2013–2014.

	OR(CI95%)	p-value
**Variables**		
**INDIVIDUAL CHARACTERISTIC OF THE ADOLESCENT**		
**Sex**		
Girls	Ref.	
Boys	1.08(**1.07–1.09**)	**<0.001**
**Age**		
12–13	Ref.	
14–15	1.77(**1.74–1.79**)	**<0.001**
16–17	2.49(**2.45–2.53**)	**<0.001**
**Self-referred skin color**		
White	Ref.	
Black	1.12(**1.10–1.14**)	**<0.001**
Mixed	1.01(**1.00–1.03**)	**<0.001**
**Residence**		
Living with both parents	Ref.	
Living with at least one parent	1.29(**1.28–1.30**)	**<0.001**
Living alone	1.40(**1.38–1.43**)	**<0.001**
**Paid job in the previous 12 months**		
No	Ref.	
Yes	1.68(**1.65–1.71**)	**<0.001**
**Mother’s schooling**		
Major degree	Ref.	
Complete High School	0.98(0.97–1.00)	0.074
Complete Middle School	1.02(**1.01–1.04**)	**0;001**
Elementary School or Illiterate	0.92(**0.91–0.94**)	**<0.001**
**Intake of meals provided by the school**		
Yes	Ref.	
No	1.12(**1.11–1.13**)	**<0.001**
**Common Mental Disorders**		
No	Ref.	
Yes	1.59(**1.58–1.61**)	**<0.001**
**SCHOOL ENVIRONMENT**		
**Capital**		
No	Ref.	
Yes	1.10 (**1.08–1.13**)	**<0.001**
**Management type**		
Public	Ref.	
Private	1.29(1.26–1.32)	**<0.001**
**Economically favored Brazilian region**		
No (Northern and Northeastern)	Ref.	
Yes (Southern, Southeastern and Midwestern)	1.56 (**1.53–1.59**)	**<0.001**
**Food selling**		
No	Ref.	
Yes	1.04(**1.02–1.07**)	**<0.001**
**Food provided by the school**		
No	Ref.	
Yes	1.01(0.98–1.04)	0.275

Table 4 presents the Multilevel Logistics Regression Model for degree of belonging to CVD-risk profile. Model 1, based on adolescent-level variables, has shown that meeting positive criterion for CMD and not consuming the meals provided by the school have significantly increased the likelihood of belonging to the CVD-risk profile (Table 5).

**Table 5:** Adjusted multilevel logistic regression model (OR and p-value) of individual characteristic of the adolescent and school environment according to profiles generated for the coexistence of risk factors for cardiovascular diseases among Brazilian adolescents–ERICA, Brazil.

	Null Model		Model 1*		Model 2**	
	OR(CI95%)	p-value	OR(CI95%)	p-value	OR(CI95%)	p-value
Variables						
**INDIVIDUAL CHARACTERISTIC OF THE ADOLESCENT**						
**Intake of meals provided by the school**						
Yes			Ref.		Ref.	
No			1.09(**1.08–1.10**)	**<0.001**	1.09(**1.07–1.10**)	**<0.001**
**Common Mental Disorders**						
No			Ref.		Ref.	
Yes			1.52(**1.50–1.54**)	**<0.001**	1.52(**1.50–1.54**)	**<0.001**
**SCHOOL ENVIRONMENT**						
**Management type**						
** Public**			-		Ref.	
** Private**			-		1.14(**1.08–1.21**)	**<0.001**
**Economically favored Brazilian region**						
** No (Northern and Northeastern)**			-		Ref.	
** Yes (Southern, Southeastern and Midwestern)**			-		1.64 (**1.60–1.67**)	**<0.001**
** Fixed Effect**						
** Intercept**	0.41 (**0.40–0.42**)	**<0,001**	0.16 (**0.15–0.17**)	**<0.001**	0.14(**0.13–0.15**)	**<0.001**
**Random effect**						
**Variance**	0.23(0.003)		0.27(0.004)		0.20(0.004)	
**Variance reduction %**					13,63%	
**Variance Partition Coefficient (VPC)**	0.067		0.075		0.059	
**AIC**	1292760		935415.2		886132.7	

Note: * Adjusted by sex, age, self-referred skin color, living with parents, paid job and mother’s schooling.

**Adjusted for variables in model 1 and added with school location (capital or not), food selling inside or around the school, and food provided by the school.

After the school variables were included in the model (Model 2), it was possible observing that adolescent-level variables maintained the associations in the same directions of those in Model 1. Studying in private schools and living in economically favored Brazilian regions have increased adolescents’ likelihood of belonging to the CVD-risk profile.

Variance between schools has decreased by 13.63%, after the inclusion of adolescents and schools’ variables in models 1 and 2. This outcome has suggested that variables included in model 2 have contributed to explain the variability in the degree of belonging to the CVD-risk profile.

## Discussion

Results in the current cross-sectional study have shown that Brazilian adolescents presented more than one risk factor for CVD (smoking habit, alcohol intake and UPF intake (≥ 80.00% of TEV)) at the same time; the weighted prevalence of risk factor reached 30.46% and 28.35% among girls and boys, respectively. Multilevel Logistics Regression analysis has shown that individuals presenting positive CMD criterion, who did not eat the food provided by the school, who studied in private schools and who lived in economically favored Brazilian regions were quite likely to belong to the CVD-risk profile.

The coexistence of risk factors observed in the current study was also recorded in other studies conducted in Brazil, which also presented weighted prevalence for the coexistence profile of CVD-risk factors similar to the current findings. Ricardo et al. (2019) [15] have analyzed data about 101,607 adolescents, extracted from the Brazilian National Survey of School Health (PeNSE). They found that 83% of adolescents accumulated two, or more, non-communicable diseases (NCD)-risk factors. Jardim et al. (2018) [32] have analyzed 1,170 students from the Brazilian Midwestern region and found that most of them presented at least two risk factors (68.9%) and that more than 10% of them presented more than four risk factors for NCD. According to Nunes et al. (2016) [33], 9 out of 10 adolescents from Southern Brazil presented two, or more, risk factors for NCD. It is important highlighting that Jardim et al. (2018) [32] and Nunes et al. (2016) [33] have conducted studies in economically favored Brazilian regions.

The coexistence of modifiable risk factors is not a reality exclusive to Brazil. A study carried out with adolescents in Canada has shown that 65% of them had two, or more, risk factors such as alcohol intake and tobacco smoking [34]. Another study, also carried out in Canada, has identified risk profile similar to the one found in the current study, due to tobacco smoking, as well as to alcohol, fast food and sugary drinks intake [35].

A cohort study that followed-up the Norwegian adolescent population until they reached adulthood has found that the investigated group can follow three likely paths, one of them was similar to the risk profile observed in the current study, called “unhealthy path” [36]. According to this path, adolescents had the habit of smoking, drinking alcohol and adopting fruit-poor diets. It is worth emphasizing that when these habits were acquired during adolescence, they remained in adulthood [36].

“Belonging to economically favored Brazilian regions” has significantly increased the likelihood of belonging to the CVD-risk profile. This outcome has suggested that the socio-economic characteristic of adolescents’ region and, consequently, their family income may play key role in determining these factors. Wang and Wang (2020) [37] have shown that mortality rates due to NCD have decreased due to economic development, i.e., NCDs were effectively controlled by the socio-economic development level of the country. This association can also be attributed to the fast globalization process [37,38], which can increase individuals’ access to modifiable risk factors, such as unhealthy foods, alcohol intake and smoking habit, and increase CVD prevalence [3,38].

Adolescents’ diet was analyzed in several studies focused on investigating the coexistence of modifiable risk factors, as well as in the current study, which focused on evaluating UPF intake among Brazilian adolescents. Data deriving from the Family Budget Survey (POF) conducted in Brazil from 2017 to 2018 have shown that UPF accounts for approximately 26.7% of total daily calories consumed by adolescents, on average [39]. Studies have focused on investigating how obesogenic food consumed in school environments can be, by taking into consideration unwanted UPF intake rates, scientific evidence about the harm caused by this food type [40–42] and the long time spent by adolescents at schools (more than one third of the day) [43].

Studies have shown difference in food environment between Brazilian private and public schools—food environment in private schools is more obesogenic than that of public schools [44–46]. This difference in Brazil is justified by the fact that public schools are instructed by Ministry of Education to follow the National School Feeding Program (PNAE), according to which, schools must provide healthy meals for free, as well as nutrition education, to students. The aforementioned program also recommends avoiding to have cafeterias inside schools. Private schools do not count on national regulation about UPF selling, and it may be associated with the obesogenic potential of the food environment observed in these schools [46].

Results in the current study have shown that not eating meals provided by schools, studying in private schools and living in socioeconomically developed Brazilian regions significantly increase adolescents’ likelihood of belonging to the CVD-risk profile. This outcome highlights the importance of including factors associated with food environment in schools in discussions about CVD prevention among adolescents.

The innovative features of the current study is on the fact that it included CMDs as variable in the analyses, which indicated that individuals presenting positive CMD criterion have significantly increased their likelihood of belonging to the CVD-risk profile. CMDs have been associated with the abuse of substances such as alcohol and tobacco [47,48], as well as with adolescents’ socioeconomic conditions [49] and gender issues [50], which predispose them to experience mental suffering and to act as risk factors for NCD [51].

Sample representativeness is another strong feature of the present study, since it has external validity and allows extrapolating the results to the Brazilian population of adolescents in the age group 12–17 years. However, it is necessary addressing some limitations of this study, such as the "social desirability" bias, i.e., adolescents’ likely trend to respond to the questionnaire based on previously standardized and well accepted social behaviors. However, adolescents were informed about the anonymity of their responses. ERICA adopted anonymity of their responses by taking into consideration self-reported behaviors, which may have led to information bias and have likely underestimated the prevalence of risk behaviors in the investigated population. In addition, the use of 24-hour dietary recall was also a limitation in the current study, since it may not have represented participants’ usual food intake.

## Conclusions

The coexistence of risk factors for CVD was observed in Brazilian adolescents whose behavioral patterns encompassed risk factors such as smoking, alcohol intake and UPF intake. Based on the analysis applied to school environment variables, it was possible seeing increased likelihood of coexistence of risk factors for CVD in adolescents studying in private schools located in socioeconomic developed Brazilian regions. Moreover, adolescents who did not eat the food provided by schools and who presented positive CMT criteria were more likely to belong to the profile associated with coexistence of risk factors for CVD.

The present study represents an advancement in the process of identifying the coexistence of risk factors for CVD in Brazil. In addition, it was the first research conducted with data deriving from ERICA in order to identify these profiles. It also helped improving the process to identify the influence of variables, be them individual or associated with the school context of Brazilian adolescents for CVD. Finally, it can be used to substantiate the inclusion of food environment variables in public policies focused on preventing CVD development among Brazilian adolescents.

In light of the foregoing, it is recommended adopting strategies based on multiple components to enable more effective interventions to prevent CVD risk factors. It is worth emphasizing that the school environment is also a place capable of influencing adolescents’ behavior. Thus, this environment should to be better controlled by regulating the sale of ultra-processed food, mainly to private schools.
